# A Pathovar of *Xanthomonas oryzae* Infecting Wild Grasses Provides Insight Into the Evolution of Pathogenicity in Rice Agroecosystems

**DOI:** 10.3389/fpls.2019.00507

**Published:** 2019-04-30

**Authors:** Jillian M. Lang, Alvaro L. Pérez-Quintero, Ralf Koebnik, Elysa DuCharme, Soungalo Sarra, Hinda Doucoure, Ibrahim Keita, Janet Ziegle, Jonathan M. Jacobs, Ricardo Oliva, Ousmane Koita, Boris Szurek, Valérie Verdier, Jan E. Leach

**Affiliations:** ^1^Department of Bioagricultural Sciences and Pest Management, Colorado State University, Fort Collins, CO, United States; ^2^IRD, Cirad, Univ. Montpellier, IPME, Montpellier, France; ^3^Centre Régional de Recherche Agronomique de Niono, Institut d’Economie Rural, Bamako, Mali; ^4^Laboratoire de Biologie Moléculaire Appliquée, Université des Sciences Techniques et Technologiques de Bamako, Bamako, Mali; ^5^Pacific Biosciences, Menlo Park, CA, United States; ^6^Department of Plant Pathology, Infectious Disease Institute, Ohio State University, Columbus, OH, United States; ^7^International Rice Research Institute, Los Baños, Philippines

**Keywords:** *Xanthomonas oryzae*, transcription activator-like effectors (TALEs), agroecosystem, cutgrass, rice

## Abstract

*Xanthomonas oryzae* (*Xo*) are globally important rice pathogens. Virulent lineages from Africa and Asia and less virulent strains from the United States have been well characterized. *Xanthomonas campestris* pv. *leersiae* (*Xcl*), first described in 1957, causes bacterial streak on the perennial grass, *Leersia hexandra*, and is a close relative of *Xo*. *L. hexandra*, a member of the Poaceae, is highly similar to rice phylogenetically, is globally ubiquitous around rice paddies, and is a reservoir of pathogenic *Xo*. We used long read, single molecule real time (SMRT) genome sequences of five strains of *Xcl* from Burkina Faso, China, Mali, and Uganda to determine the genetic relatedness of this organism with *Xo*. Novel transcription activator-like effectors (TALEs) were discovered in all five strains of *Xcl*. Predicted TALE target sequences were identified in the *Leersia perrieri* genome and compared to rice susceptibility gene homologs. Pathogenicity screening on *L. hexandra* and diverse rice cultivars confirmed that *Xcl* are able to colonize rice and produce weak but not progressive symptoms. Overall, based on average nucleotide identity (ANI), type III (T3) effector repertoires, and disease phenotype, we propose to rename *Xcl* to *X. oryzae* pv. *leersiae* (*Xol*) and use this parallel system to improve understanding of the evolution of bacterial pathogenicity in rice agroecosystems.

## Introduction

Rice is a staple crop for more than half the world. Severe rice diseases, such as bacterial leaf streak (BLS) caused by *Xanthomonas oryzae* pv. *oryzicola* (*Xoc*) and bacterial blight (BB), caused by *X. o.* pv. *oryzae* (*Xoo*), are increasing in prevalence in parts of Asia and sub-Saharan Africa and cause significant yield losses. In Asia, perennial weeds are considered an important source of primary pathogen inoculum for these two diseases (Ou, 1985; Mew, 1987).

Southern cutgrass (*Leersia hexandra* Swartz) is a common grass found in the southern United States, South America, Africa, and Asia. It is a member of the Poaceae family and is closely related to rice, but diverged from *Oryza* approximately 14 mya (Guo and Ge, 2005). *L. hexandra* is an invasive species that frequently grows along rivers and canals surrounding rice paddies. Because of its close relationship to *Oryza* spp., *Leersia* spp. are included as outgroups in phylogenetic studies. Recent genome investigations of *Leersia perrieri*, a cutgrass found in Madagascar, were done to compare repetitive elements and transposable elements among *Oryza* sp. and to uncover orthologs of the important submergence tolerance gene, *SUB1* (Copetti, 2013; Copetti et al., 2015; dos Santos et al., 2017). One-third of the *L. perrieri* genome was found to consist of repeats. The high amount of newly discovered repeats (35%) indicates that the *L. perrieri* genome is evolving rapidly relative to the *Oryza* genus (Copetti, 2013).

Early reports of phytoremediation by *L. hexandra* showed this grass’ capacity to sequester Cr, Cu, and Ni, and it has now been proposed as a tool in wastewater treatment (Liu et al., 2011; Wang et al., 2012; You et al., 2013). Interestingly, this and other grasses in this genus, such as *Leersia sayanuka*, *Leersia oryzoides*, and *Leersia japonica*, are susceptible to *Xoo* (Noda and Yamamoto, 2008) and can serve as reservoirs for inoculum. *Xoo* strains isolated from symptomless *L. hexandra* cause BB symptoms in rice, and, in artificially inoculated weed plants*, Xoo* multiplied without evidence of disease (Gonzalez et al., 1991) implicating this grass as an alternative host for the pathogen. These and other findings reinforce that effective integrated management of crop diseases must incorporate knowledge of pathogen interactions with weedy species.

The species *Xo* is highly diverse, and is represented by distinct lineages of *Xoo* from Asia and Africa, *Xoc* from Asia and Africa, and strains not assigned as a pathovar from the United States, *Xo* (Gonzalez et al., 2007; Triplett et al., 2011; Hajri et al., 2012; Poulin et al., 2015; Triplett and Leach, 2016). The term pathovar is used to refer to a strain or set of strains with the same or similar characteristics, differentiated at infrasubspecific level from other strains of the same species or subspecies on the basis of distinctive pathogenicity to one or more plant hosts^1^. Poulin et al. (2015) used multi-locus variable-number tandem-repeat analysis (MLVA) to investigate genetic structures of microbial populations (Zhao et al., 2012; Poulin et al., 2015), and suggested that *Xoo* and *Xoc* from Africa had a common Asian ancestor; this conclusion was based on the fact that the allelic richness, or number of alleles, was significantly less in these populations. However, further analyses on an extensively sampled set of isolates are needed to confirm this ancestral hypothesis.

*Xanthomonas* spp. inject effector proteins into plant host cells to elicit disease via a type-III (T3) secretion system (White et al., 2009). These proteins can confer pathogenicity and/or dictate host specificity (Jacques et al., 2016). *Xanthomonas* spp. are most notable for production of transcription activator-like effectors (TALEs). TALEs influence host gene expression by directly binding to specific sequences [effector binding elements (EBEs)] in the target promoter as dictated by repeat-variable di-residues (RVDs) at the 12 and 13 amino acid position in the central repeat region (CRR) (Boch et al., 2009; Moscou and Bogdanove, 2009; White et al., 2009; Bogdanove and Voytas, 2011). The CRR contains different numbers of repeats, each with 33–35 amino acids. TALEs may enhance diseases by targeting susceptibility (S) genes, or may trigger a resistance response through activation of an “executor” resistance gene (R) expression (Boch et al., 2014; Hutin et al., 2015).

The presence of TALE effectors is variable in the genus (Jacques et al., 2016). *Xoo* contain nine to 20 TALEs while *Xoc* can contain up to 29. US *Xo* do not contain any TALEs, and due to this absence, have been employed as a tool to study TALE effector biology in rice (Ryba-White et al., 1995; Verdier et al., 2012). New sequencing technologies and predictive algorithms have accelerated the characterization of TALEs and their host gene targets. In particular, long read, single molecule real time (SMRT) sequencing (Pacific Biosciences, Menlo Park, CA, United States) has enabled the rapid assembly of TALE sequences that are otherwise laborious to capture due to their highly repetitive structure (Eid et al., 2009; Booher et al., 2015; Wilkins et al., 2015; Grau et al., 2016; Peng et al., 2016; Quibod et al., 2016; Tran et al., 2018). Collectively, TALE repertoires (TALomes) encoding polymorphic groups that have contrasted abilities to induce susceptibility target genes potentially underlie host adaptation at a small evolutionary scale (Doucouré et al., 2018). The *Xo* group has clearly undergone significant evolution influenced by geography, environment, and host, and TALomes can provide critical insight into how these events occur.

*Xanthomonas campestris* pv. *leersiae* (*Xcl*), a pathogen of *L. hexandra*, was previously shown to group distinctly from *Xo* by host range and phylogenetic analysis (Fang et al., 1957; Parkinson et al., 2009). However, using a multi-locus sequence alignment (MLSA) analysis Triplett et al. (2011) showed that *Xcl* strain NCPPB4346, which was isolated from southern cutgrass in China, groups within the *Xo* cluster, yet it could not be assigned to any described pathovar. A more recently isolated strain, BAI23 from weeds in Burkina Faso, showed high sequence similarity with *Xcl* NCPPB4346 based on a MLSA analysis as well as the presence of TALEs (Wonni et al., 2014). Together, the two strains form a distinct genetic cluster within *X. oryzae.*

Prediction algorithms based on the TALE’s specific RVD pattern and their corresponding degenerate DNA code has facilitated identification of plant target genes whose promoters contain EBEs for TALE binding (Doyle et al., 2012; Grau et al., 2013, 2016; Pérez-Quintero et al., 2013; Booher and Bogdanove, 2014; Yang et al., 2014). Many *S* genes are transporters (sugar or sulfate) or transcription factors and upon induction facilitate bacterial colonization and symptom development (Hutin et al., 2015; Tran et al., 2018). Although a large body of work is available on TALEs from *Xoo* and *Xoc*, no information has been reported on the TALEs from *Xcl*, how they compare to those in other *X. oryzae*, and the nature of their predicted targets within *Leersia* spp. In this study, we used comparative genomics, identification of T3 effectors, TALomes, and disease phenotyping to characterize *Xcl.* We used gene target prediction algorithms to identify potential *Xcl* TALE gene targets in draft *Leersia* genome sequences. Finally, we provide evidence to support renaming *Xcl* to *X. oryzae* pv. *leersiae* (Fang et al., 1957) and will refer to this organism as *Xol* throughout this work.

**Table 1:** Bacterial strains used in phenotyping and molecular diagnostics.

Species	Strain	Origin	Host	Reference or source	Response to diagnostic primer set	
					Xol5	Xol7
*Burkholderia andropogonis*	3549		*Zea mays*	L.E. Claflin	−	−
*Escherichia coli*	DH5α				−	−
*Pseudomonas fuscovaginae*	SE-1	Philippines	*Oryza sativa*	G. Ash	−	−
*P. syringae* pv. *syringae*	M108	United States	*Solanum lycopersicum*	H.F. Schwartz	−	−
*Xanthomonas* sp.	M136	Mali	*O. sativa*	V. Verdier	−	−
*Xanthomonas* sp.	SHU100	Philippines	*O. sativa* seed	C.M. Vera Cruz	−	−
*X. campestris* pv. *alfalfae*	KX-1	United States		L.E. Claflin	−	−
*X. c.* pv. *campestris*	X1910	United States	*Brassica oleracea*	N. Dunlop	−	−
*X. c.* pv. *pelargonii*	X5	United States	*Geranium* sp.	L.E. Claflin	−	−
*X. euvesicatoria*	85-10	United States	*Capsicum frutescens*	A. Bogdanove	−	−
*X. euvesicatoria*	O177	United States	*Allium cepa*	H.F. Schwartz	−	−
*X. oryzae*	X11-5A^a^	United States	*O. sativa*	Triplett et al., 2011	−	−
*X. o.* pv. *leersiae*	BAI23^a^	Burkina Faso	Weeds	V. Verdier, Wonni et al., 2014	+	+
*X. o.* pv. *leersiae*	BB 151-3	Uganda	*O. sativa*	B. Yang, R. Oliva, G. Onaga	+	+
*X. o.* pv. *leersiae*	BB 156-2	Uganda	*O. sativa*	B. Yang, R. Oliva, G. Onaga	+	+
*X. o.* pv. *leersiae*	NCPPB4346^a^	China	*Leersia hexandra*	Triplett et al., 2011; Wonni et al., 2014	+	+
*X. o.* pv. *leersiae*	NJ 6.1.1	Mali	*L. hexandra*	This study	+	+
*X. o.* pv. *oryzae*	A3857	India	*O. sativa*	J.E. Leach	−	−
*X. o.* pv. *oryzae*	BAI3^a^	Burkina Faso	*O. sativa*	V. Verdier	−	−
*X. o.* pv. *oryzae*	MAI1	Mali	*O. sativa*	V. Verdier	−	−
*X. o.* pv. *oryzae*	NAI8	Niger	*O. sativa*	V. Verdier	−	−
*X. o.* pv. *oryzae*	PXO86	Philippines	*O. sativa*	C.M. Vera Cruz	−	−
*X. o.* pv. *oryzae*	PXO99^Aa^	Philippines	*O. sativa*	J.E. Leach	−	−
*X. o.* pv. *oryzae*	Xoo4	Thailand	*O. sativa*	J.E. Leach	−	−
*X. o.* pv. *oryzae*	Xoo199	Korea	*O. sativa*	S.H. Choi	−	−
*X. o.* pv. *oryzicola*	BLS98	Philippines	*O. sativa*	C.M. Vera Cruz	−	−
*X. o.* pv. *oryzicola*	BLS105	Philippines	*O. sativa*	C.M. Vera Cruz	−	−
*X. o.* pv. *oryzicola*	BLS256^a^	Philippines	*O. sativa*	A. Bogdanove	−	−
*X. o.* pv. *oryzicola*	BLS305	Philippines	*O. sativa*	C.M. Vera Cruz	−	−
*X. o*. pv. *oryzicola*	MAI4	Mali	*O. sativa*	V. Verdier	−	−
*X. o.* pv. *oryzicola*	MAI10^a^	Mali	*O. sativa*	V. Verdier	−	−
*X. translucens*	LH2-1	United States	*L. hexandra*	This study	−	−
*X. t.* pv. *cerealis*	NCPPB1944	United States	*Bromus inermis*	V. Verdier	−	−
*X. t.* pv. *phleipratensis*	PDDCC5744	United States	*Phleum pretense*	C. Stevens	−	−
*X. t.* pv. *translucens*	B76	United States	*Hordeum vulgare*	N. Tisserat	−	−
*X. t.* pv. *translucens*	NCPPB2389	India	*H. vulgare*	C. Bragard	−	−
*X. t.* pv. *translucens*	UPB787	Paraguay	*H. vulgare*	C. Bragard	−	−
*X. t.* pv. *undulosa*	UPB513	Mexico	*T. aestivum*	C. Bragard	−	−

*^a^Strains tested for pathogenicity to Oryza sativa and Leersia hexandra.*

## Materials and Methods

### Bacterial Strains and Plant Varieties

Bacterial strains included in this study are listed in Table 1. Bacteria were cultured on peptone sucrose agar (PSA) at 28°C for plant inoculations. Genomic DNA for sequencing was isolated from *Xol* strains BAI23, BB 151-3, BB 156-2, NCPPB4346, and NJ 6.1.1 grown for 48 h on nutrient agar at 28°C (Lang et al., 2014; Wonni et al., 2014). Barley (*Hordeum vulgare* L. cultivar Morex), wheat (*Triticum aestivum* cv. Chinese Spring) and tobacco (*Nicotiana tabacum*) were grown in a growth chamber at 22°C, 50% relative humidity, and 16 h of light. Rice (*Oryza sativa*) varieties included in pathogenicity assays were Azucena, Carolina Gold, Cypress, IR64, and Nipponbare.

Southern cutgrass (*L. hexandra)* was collected in Texas, United States, and seed was propagated at Colorado State University. The seed was scarified then germinated in porous ceramic silica (Greens Grade, Profile Products, LLC, Buffalo Grove, IL, United States) and 0.5x Hoagland’s solution with the following modifications: 2.5 mM KNO_3_, 1 mM MgSO_4_, 3 mM KH_2_PO_4_, 2.5 Ca(NO_3_)_2_, 0.05 mM FeSO_4_, and 0.1 mM (Na)_2_EDTA (Hoagland and Arnon, 1950). Seed was incubated in a petri dish in the dark for 8 days, then in the light for 30 days at 28°C. Germinated seeds were transplanted into 1-gallon pots with equal parts Greens Grade and ProMix BX (ProMix, Quakertown, PA, United States), and grown in a greenhouse (27 ± 1°C, 16 h day length, and 80–85% relative humidity). Additional plants were obtained from the two mother plants via rhizome propagation. Propagation began 30 days after planting with subsequent propagation every week to allow time for new growth. To promote root growth, plants were placed in the dark for 24 h.

### Pathogenicity Assays

Rice varieties were inoculated at 4–5 week-old with suspensions (10^8^ CFU mL^−1^) of bacterial strains listed in Table 1. Bacterial suspensions were both infiltrated into the intercellular spaces of rice leaves on either side of the main vein with a needleless syringe and inoculated by leaf clipping as described (Kauffman et al., 1973; Reimers and Leach, 1991). Two leaves were inoculated on each of three to six separate plants; water was included as a negative control. The entire experiment was conducted twice. Lesions were measured at 12 days post inoculation (dpi), and bacterial numbers *in planta* were quantified as previously described (Verdier et al., 2012).

### Molecular Diagnostics

To test relationships of *Xol* to *Xo* (Notomi et al., 2000; Triplett et al., 2011; Wonni et al., 2014), a diagnostic multiplex and loop mediated isothermal amplification (LAMP) PCR were used (Lang et al., 2010, 2014; Wonni et al., 2014). Previously described universal US *Xo* primers were also tested to differentiate *Xol* from this novel US clade within the species (Triplett et al., 2011). UniqPrimer was employed to compare draft *Xol* genomes (BAI23 and NCPPB4346) and generate primers specific to *Xol* as previously described (Ash et al., 2014; Lang et al., 2014, 2017; Juanillas et al., 2018). Specificity was validated by screening *Xol* primers against diverse pools of bacterial genomic DNA (Table 1). Primers, expected product size, and optimal annealing temperatures are listed in Supplementary Table S1.

### Genome Sequencing and Assembly

Long read, SMRT sequencing (PacBio, Menlo Park, CA, United States) data were generated for five *Xol* strains and for the *Xo* strain X11-5A, to be used as an outgroup. DNA for SMRT sequencing was isolated by culturing strains on nutrient agar for 48 h then using the Genomic DNA buffer set and Genomic-tips according manufacturer instructions (Qiagen, Valencia, CA, United States). SMRT sequence was assembled using HGAP v4 (PacBio, Menlo Park, CA, United States). Genomes were circularized using circulator (Hunt et al., 2015). Assemblies and raw data have been deposited in NCBI (BioProject IDs PRJNA522807 and PRJNA522811; BioSample accessions SAMN03862116, SAMN02469650, SAMN10956066-68, SAMN10956070; raw sequencing files SRX5417793-98; Assembly accessions CP036251-56). Assembly CP036251 (X11-5A) replaces draft assembly GCF000212755.1 and assembly CP036253 (NCPPB4346) replaces draft assembly GCF001276975.1 (also in Bioproject PRJNA257008). Accessions for all genomes used in this study are listed in Supplementary Table S2.

### Phylogenomics and Bioinformatic Analyses

In addition to the five *Xol* sequenced strains, all completely sequenced *X. oryzae* genomes were obtained from the NCBI to be used for comparisons, as well as representative genomes from other *Xanthomonas* species. All genomes and accessions can be found in Supplementary Table S2.

Average nucleotide identity (ANI) values were obtained using the ANI-matrix script from the enveomics collection (v1.3) (Rodriguez-R and Konstantinidis, 2016). Parsimony trees based on pan-genome SNPs were obtained using KSNP (v3.0) (Gardner et al., 2015). Multi-locus sequence analysis was made by identifying 33 housekeeping genes in all genome using Amphora2 (Wu and Scott, 2012). Concatenated amino acid sequences of these genes were then aligned using MUSCLE (v3.8.31) (Edgar, 2004), and neighbor-joining bootstrapped trees were generated using functions (dist.ml; model = “Blosum62”, NJ, bootstrap.pml) of the R package phangorn (v2.4.0) (Schliep, 2011).

Automated annotation of proteins used in this manuscript for all genomes was made using Prokka (v1.14-dev) (Seemann, 2014), annotation for public versions available in the NCBI was made with the NCBI Prokaryotic Genome Annotation Pipeline (PGAP). Groups of orthologs and trees based on presence/absence of ortholog genes were generated using OrthoFinder (v 2.2.6) (Emms and Kelly, 2015). Dotplots of whole genome alignments were obtained using Gepard (v. 1.4, using word length 100) (Krumsiek et al., 2007). Genome duplications were then quantified using minimap (v.1) (Li, 2016) as implemented in minidot^2^ (parameters = −g 1000 −k 50 −w 5 −L 100). Colinear gene regions were identified using DAGchainer (Haas et al., 2004) (parameters = −E 1e-20 −s −g 2000 −x 200 −A 4) using the blast results from orthofinder as inputs. Insertion sequences (ISs) were identified using ISEScan (v1.6) (Xie and Tang, 2017).

Non-TALE T3 effectors were identified by BLASTP (v. 2.6.0+, results were filtered keeping hits with −evalue < 0.0001, > 0% identity in >40% the query length) (Boratyn et al., 2013) of consensus effectors sequences obtained from http://xanthomonas.org/ against the protein sequences obtained using Prokka. TALE sequences were extracted from each genome using in-house Perl scripts^3^. Neighbor joining trees were generated from concatenated nucleotide TALE N and C terminal sequences, alignments were made using MUSCLE (Edgar, 2004) and trees were generated using functions (dist.ml; model = “JC”, NJ, bootstrap.pml) of the phangorn (v2.4.0) (Schliep, 2011).

Transcription activator-like effector repeat sequences were aligned using DisTAL v1.2 (Pérez-Quintero et al., 2015) and neighbor joining trees were generated based on DisTAL genetic distances using the R package ape (Paradis et al., 2004). TALE groups were defined using the function cutree in R (height = 4.8) on the DisTAL tree. Predictions for TALE binding sites were made using Talvez on the promoters (−1 kb upstream of the translation start site) of annotated genes in the *L. perrieri* (v1.4) and *O. sativa* cv. Nipponbare (v. MSU7) as previously described (Pérez-Quintero et al., 2013).

## Results

### *Xol* Is Pathogenic to Rice and Southern Cutgrass

**FIGURE 1:**
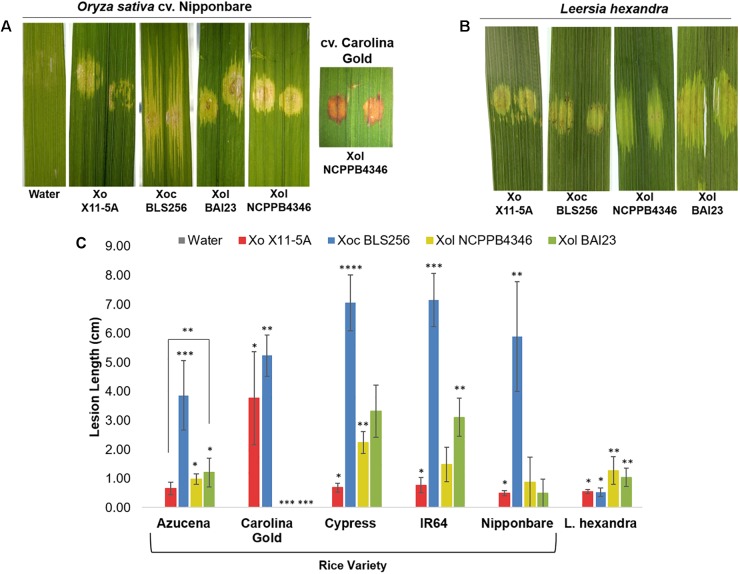
*X. oryzae* (*campestris*) pv. *leersiae* (Xcl) and *X. oryzae* cause water soaking on reciprocal hosts, but virulence is highest on original host. Phenotypes and quantitative lesion lengths caused by *Xanthomonas oryzae* (Xo) X11-5A, *X. o.* pv. *oryzicola* (Xoc) BLS256 and *X. o.* pv. *leersiae* NCPPB4346 on **(A,C)** diverse rice varieties and **(B,C)** wild *Leersia hexandra* were measured 12 days post infiltration inoculation. An asterisk denotes a significant difference between strains on each variety (*p* ≤ 0.05). Error bars represent ± SD.

To better understand the biology of *Xol*, we established a host range by screening for pathogenicity on rice and southern cutgrass (*L. hexandra*) using different inoculation techniques. Relative to the virulent Philippine *Xoc* strain BLS256, the *Xol* strains were less aggressive to rice, and caused less expansive water-soaked leaf streaking on several rice varieties. *Xol* BAI23 was more aggressive than NCPPB4346 on rice varieties Cypress and IR64, and caused more disease than the US *Xo* strain X11-5A on Azucena. Rice variety Carolina Gold was resistant to *Xol*, exhibiting a hypersensitive response and no lesion expansion after infiltration. Both *Xol* strains caused longer lesions on southern cutgrass than *Xo*, but were not as aggressive as *Xoc* BLS256 on rice. *Xo* produced water-soaked spots at the point of infiltration that did not expand on southern cutgrass (Figure 1).

**FIGURE 2:**
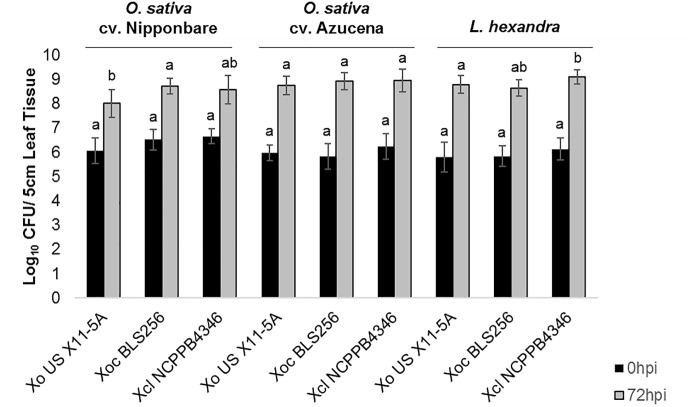
*X. oryzae* pv. *leersiae* can grow and colonize rice as effectively as rice pathogens and rice pathogens can colonize *Leersia hexandra* as effectively as *X. oryzae* pv. *leersiae*. Bacterial population growth in leaves of rice varieties Nipponbare and Azucena and *L. hexandra* inoculated with *X. oryzae* X11-5A, *X. oryzae* pv. *oryzicola* BLS256, and *X. oryzae* pv. *leersiae* NCPPB4346 were quantified at 0 and 72 h post inoculation (hpi). Population sizes were measured in a 5 cm leaf segment infiltrated with each strain. Error bars represent ± SD of six independent leaves, and letters denote treatments significantly different from one another on each variety (*p* ≤ 0.05).

After clip inoculations, lesions caused by *Xol* did not expand on rice or southern cutgrass, unlike those caused by the vascular pathogen *Xoo* (Supplementary Figure S1). When infiltrated into leaves, populations of *Xol* were equivalent to *Xoc* BLS256 and *Xo* X11-5A on rice cvs. Nipponbare or Azucena after 72 hpi (Figure 2). On southern cutgrass, their native host, *Xol* grew to a significantly higher population than *Xo* X11-5A. *Xol* did not cause disease on wheat or barley (Supplementary Figure S2). Phenotyping *Nicotiana* species can serve as a screen for the ability of microbes, particularly *Xanthomonas* spp., to elicit a non-host resistance response (Gonzalez et al., 2007). *Xol* caused minor chlorosis, but not water soaking nor a hypersensitive response, when infiltrated into *N. tabacum*, similar to the phenotype caused by *Xoo* PXO99A and *Xoc* BLS256 while *Xoo* BAI3 and *Xo* US 11-5A caused a strong hypersensitive response at the site of infiltration on *N. tabacum* similar to prior reports (Gonzalez et al., 2007) (Supplementary Figure S2).

Our studies show that *Xol* is pathogenic to southern cutgrass and mildly pathogenic on diverse varieties of rice, but does not cause disease on barley or wheat, and that some rice varieties exhibit resistance to *Xol.* After inoculation, *Xol* causes symptoms on rice that are most similar to *Xoc*, i.e., expanding lesions when introduced into the intercellular spaces (leaf infiltration) and no spreading lesions when introduced into the xylem vessels (clipping).

### *X. campestris* pv. *leersiae* Belongs to the *X. oryzae* Species and Is Phylogenetically Close to *Xoc*

**FIGURE 3:**
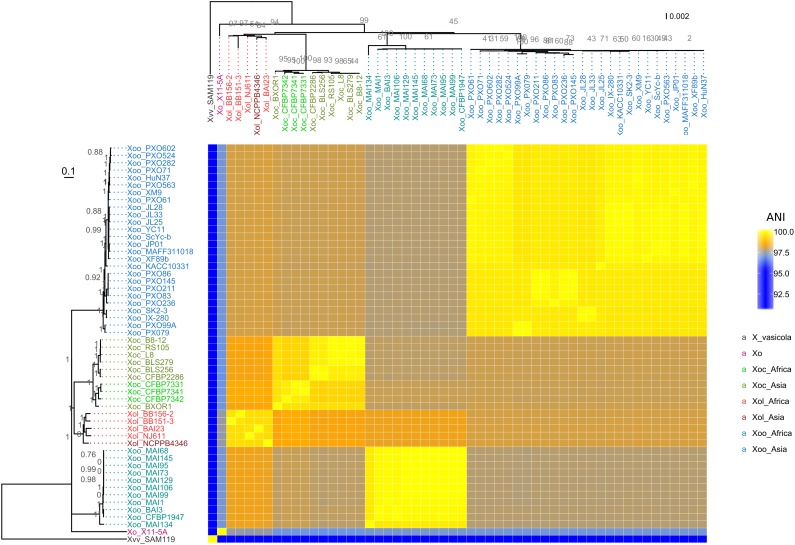
*X. oryzae* pv. *leersiae* (Xol) is a member of *X. oryzae* (Xo) and is closely related to *X. oryzae* pv. *oryzicola* (Xoc). The heatmap shows pairwise average nucleotide identity (ANI) values between fully sequenced *X. oryzae* genomes. (Left) Consensus parsimony tree generated with based on shared pangenome SNPs, numbers in gray indicate node support as outputed by kSNP3, heatmap rows are ordered according to this tree. (Top) MLSA neighbor-joining tree based on concatenated alignments of 33 housekeeping genes, numbers in gray indicate bootstrap support for branches, and heatmap columns are ordered according to this tree. *X. oryzae* pv. *oryzae* is abbreviated as Xoo and *X. vasicola* pv. *vasculorum* as Xvv. All species abbreviations are followed by strain name.

Amplification of *Xol* DNA with primers specific for *Xo* but not *Xoc*, *Xoo*, or US *Xo* suggested that *Xol* were related to *X. oryzae*, but distinct from other *Xo* pathovars (Lang et al., 2010; Triplett et al., 2011) (Supplementary Figure S3). We then used SMRT sequencing technology to derive complete genomes of five available *Xol* strains from China (NCPPB4346), Burkina Faso (BAI23), Mali (NJ611), and Uganda (BB151-3 and BB156-2). We calculated pairwise ANI among these and all fully sequenced *X. oryzae* genomes (Rodriguez-R and Konstantinidis, 2016). This analysis showed that *Xol* strains were 99–100% identical to one another, and were 97–99% identical to US *Xo*, *Xoc*, and *Xoo. Xol* were most similar to *Xoc* (∼98.5%). They were 76–91% similar to other *Xanthomonas* species. *X. vasicola* was the next most similar *Xanthomonas* species to *oryzae*, sharing 91% ANI with *Xol* and all *Xo* (Figure 3).We generated parsimony phylogenetic trees based on pan-genome SNPs using kSNP3 (Gardner et al., 2015), which showed again that *Xol* strains were closely related to *Xoc* (Figure 3). Neighbor-joining trees generated based on MLSA and on presence/absence of ortholog families showed similar groupings, although the placement of African *Xoo* in the tree was variable (Figure 3 and Supplementary Figure S4).

These combined data indicate that our sequenced strains are more closely related to *Xo* than other *Xanthomonas* species and therefore, combined with previously reported MLSA data (Triplett et al., 2011; Wonni et al., 2014), we recommend that the formal taxonomic placement of *Xcl* be included in the species “*oryzae*.” Further biological support for this shift from host range and effector repertoires is described below.

To avoid misidentification of *Xol* as a *Xoc* or *Xoo* in future studies, genomes were compared to identify regions of specificity to base diagnostic primer design with UniqPrimer (Juanillas et al., 2018). Two primer sets were validated for specificity against over 30 closely and distantly related bacteria (Supplementary Table S1). Both primer sets consistently amplified only control *Xol* strains and did not amplify any other bacterial strain tested (Table 1).

### Rice Associated *X. oryzae* Show High Genome Plasticity Compared to *X. oryzae* pv. *leersiae*

**FIGURE 4:**
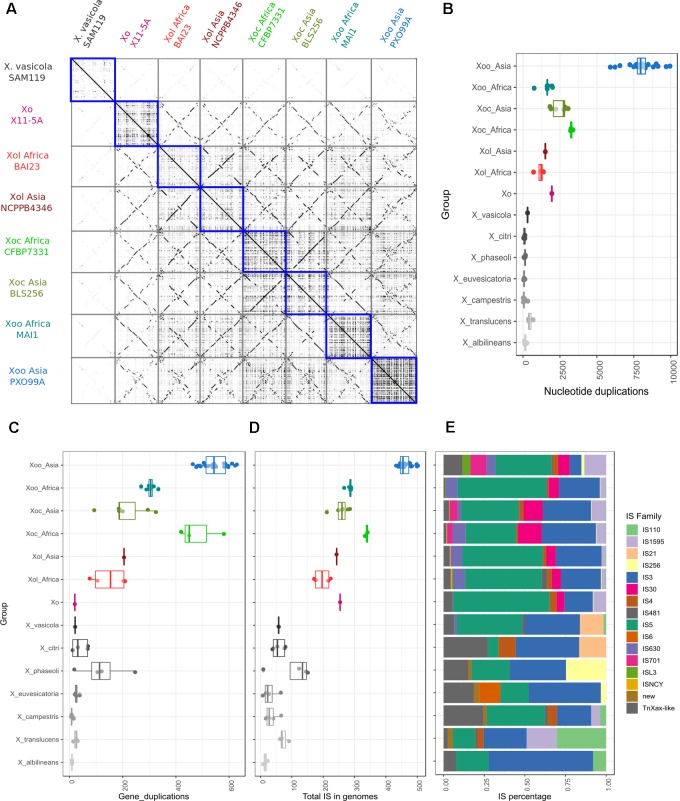
*X. oryzae* pv. *leersiae* genomes show generally less plasticity than other *X. oryzae* genomes but more than other *Xanthomonas*. **(A)** Dotplots showing pairwise whole genome alignments between representative genomes of each *X. oryzae* group, blue squares highlight self-alignments showing high amounts of duplication and rearrangements in *X. oryzae* pv. *oryzicola* and pv. *oryzae* when compared to *X. oryzae* pv. *leersiae*. **(B)** Boxplot showing total duplicated nucleotide sequences (at least 100 bp) in self-alignments of whole genomes for each group, each dot represents the total regions detected for one strain aligned to itself. **(C)** Gene duplication involving at least four co-linear genes were identified based on alignments of annotated proteins in a genome against itself, boxplot shows total duplication events detected for each strain. **(D)** Total number of insertion sequences (ISs) identified in genomes of each group. **(E)** Distribution of IS families identified within each group, percentages are calculated based on the average of each family in all strains analyzed for each group.

We generated dotplots to visualize pairwise whole genome alignments to further compare the genomes of *X. oryzae* strains. These alignments showed several genome rearrangements between the different *Xol* strains with respect to each other and to other *X. oryzae* groups. Curiously, we noticed that self-alignments of genomes of pathovars *oryzae* and *oryzicola* overall showed more genomic duplication than those of *Xol* (Figure 4A). To quantify this, we identified colinear regions within each genome that showed these possible genomic duplication events are present in different frequencies in each *X. oryzae* lineage; and that they are more frequent in *Xoc* and Asian *Xoo*, and less frequent in *Xol* and African *Xoo* (Figure 4B). Notably, all *X. oryzae* examined exhibited overall more genomic duplications than other *Xanthomonas* species (Figure 4B).

We also identified colinear arrangements of homologous genes within each genome, meaning genome duplications that involve multiple genes, and again, these were more frequent in *Xoc* and *Xoo* than in *Xol* (Figure 4C). Since some of these duplications may be mediated by duplicative transposition we annotated and quantified insertion sequences (ISs) in the different *X. oryzae* genomes, which indeed revealed a higher frequency of IS in the groups with higher amounts of duplicated regions (Figure 4). The distribution of IS families was overall similar among *X. oryzae*, with *Xol*’s most resembling the distribution of African *Xoo* (Figure 4E).

### Non-TAL T3E Repertoires of *Xol* Are Similar to *Xoc*

Computational prediction of T3E among annotated proteins in each genome revealed that the *Xol* T3E repertoire resembles more closely that of *Xoc.* Nonetheless, some features are unique to *Xol*, specifically all *Xol* strains possess a *xopD* gene that is absent in all other *Xo* genomes. *XopAH* is also present in all *Xol* strains and is shared only by two *Xoc* strains. On the other hand, *Xol* strains lack *xopO* which is present in all *Xoc* strains, and some *Xol* strains seem to lack the otherwise universal effectors *xopW and xopK* (Supplementary Figure S5).

### *Xol* Strains Have Distinct TALE Repertoires With Some Similarities to African *Xoo*

Previous Southern blot analyses using conserved TALE probes predicted that *Xol* BAI23 and NCPPB4346 had five and four TALEs, respectively (Wonni et al., 2014). However, Southern blot analysis cannot resolve TALEs that are close in size. We found in our whole genome sequences that *Xol* strains contained 12 or 13 TALEs each, which is more than the nine TALEs per genome found in African *Xoo*, and less than what is commonly found in *Xoc* (22–29 TALEs) and Asian Xoo (13–20 TALEs). No truncated TALEs were identified in any *Xol* genome. As previously reported, no TAL effectors were found in the *Xo* strain X11-5A (Ryba-White et al., 1995; Triplett et al., 2011).

**FIGURE 5:**
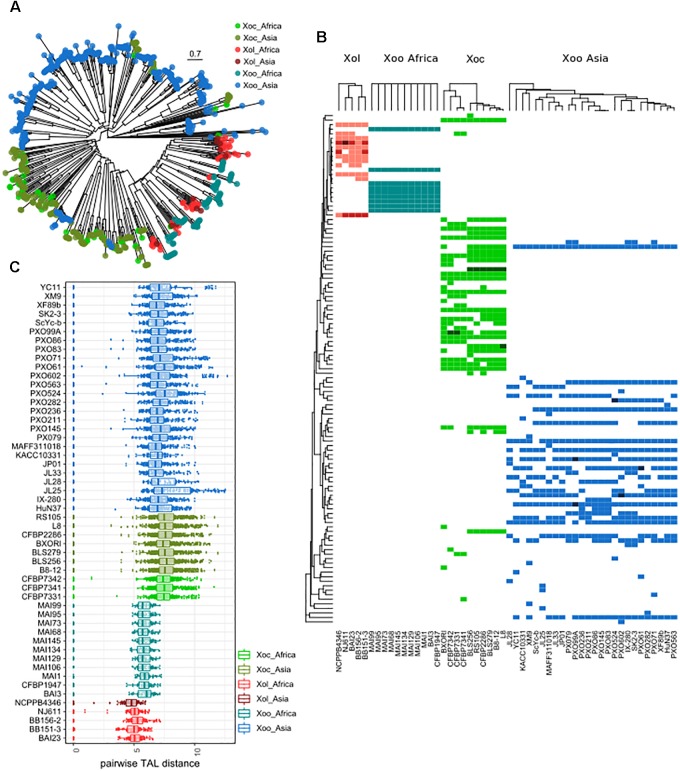
TALEs from *X. oryzae* pv. *leersiae* are unique and closely related to African *X. oryzae* pv. *oryzae* TALEs. **(A)** neighbor-joining tree based on TALE repeat arrangement alignments obtained using DisTAL. Each point represents a single TALE. **(B)** TALEs were classified in group according to the DisTAL tree, the heatmap shows presence/absence of each TALE group in *X. oryzae* strains, darker colors indicate multiple TALs from one group found in one strain. Tree to the left indicates average DisTAL distance between repeat arrangements for each TALE group. Top trees show hierarchical clustering of each *X. oryzae* group based on the presence/absence pattern of TALE groups. **(C)** Dotplots shows pairwise distance between all TALE repeat arrangements from each strain as calculated with DisTAL, each dot represents a pair of TALEs within one genome.

Phylogenetic trees based on the N and C termini of *X. oryzae* TALEs showed that *Xol* TALEs form a distinct group, but seem to be close to African *Xoo* TALEs, despite the overall genomic similarities with *Xoc* (Supplementary Figure S6). We also constructed trees based on similarities in the CRR of using DisTAL, which grouped *Xol* and African *Xoo* together in a subgroup that also includes *Xoc* (Figure 5A). We used the DisTAL tree to define TALE groups based on repeat region similarities, *Xol* TALEs were classified in 12 groups (Figure 5B). One of the groups contained TALEs from *Xoc* and *Xol* strains, while all the others were exclusive to *Xol.* Of these, seven groups were present in all five *Xol* strains. One of these groups, present in the four African *Xol* strains, contains the RVD combination “TI” which has not been previously reported in other species.

All *Xol* strains contained at least two TALEs that were classified within the same group, that is, within each *Xol* genome there are at least two TALEs with nearly identical repeat arrangements (Figure 5B). By examining pairwise genetic distances between the repeat regions of TALEs, we saw that TALEs from *Xol* are on average more similar to each other than TALEs within other *X. oryzae* groups, indicating that they are possibly less diversified and/or more redundant (Figure 5C).

### *Xol* TALE Targets in Cutgrass and Rice Are Different From *Xoc* and *Xoo* Targets

Talvez (Pérez-Quintero et al., 2013) was used to predict host targets for each TALE in our dataset in the promoters of annotated genes in the *L. perrieri* (v1.4) and *O. sativa* (vMSU7) genomes. We identified ortholog pairs between both genomes using reciprocal BLAST. Comparisons of the predictions between both genomes for *Xol* TALEs revealed very few cases where an ortholog pair was predicted to be a target in both genomes (Supplementary Figure S7), and highlighted that the promoters (and thus the targeted genes) in these two hosts are very different.

**FIGURE 6:**
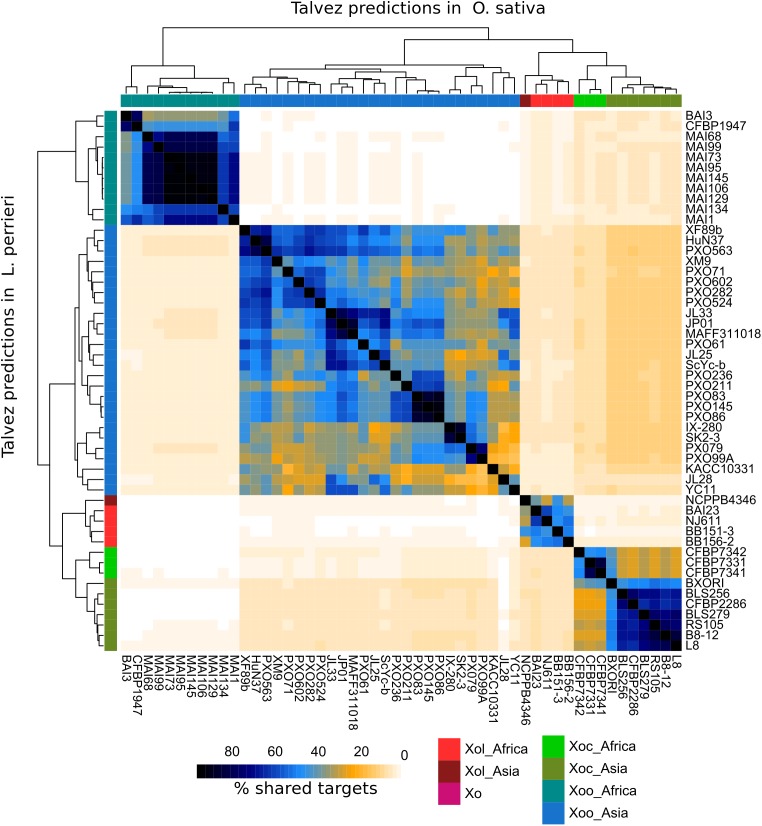
TALEs from *X. oryzae pv. leersiae* (Xol) are predicted to target distinct genes from other *X. oryzae* [Xo, *X. oryzae* pv. *oryzae* (Xoo), *X. oryzae* pv. *oryzicola* (Xoc)] strains. Predictions for binding sites were made for each TALE in the promoters (–1 kb) of annotated genes in the *L. perrieri* and *O. sativa* genomes, top 200 predicted targets for each TALE were kept, and predictions were aggregated for each strain. For each pair of strains, the overlap of predictions was calculated as the number of common genes predicted as TALE targets in both strains divided by the total genes predicted as targets in either strain, times 200 (intersect × 2 × 100/union). The lower left triangle part of the heatmap shows percentage of shared targets in the *L. perrieri* genome for each pair of strains, and the upper right shows shared targets in the *O. sativa* genome. Trees at the top and left show hierarchical clustering based on stared targets in each genome. Accession numbers for genomes used in this analysis are available in Supplementary Table S2.

We then compared predictions for all *Xol* lineages in both genomes and calculated the overlap between predictions for each strain. As a result, we saw that each *X. oryzae* group has a distinct group of predicted targets with relatively little overlap with other groups. In the case of *Xol*, the highest overlap was found with predictions for *Xoc* TALEs (10–11% shared predicted targets) (Figure 6). Given the differences in the genomes of their hosts and that *Xol* TALEs have unique repeat sequences, we expect their targets to be likewise unique. Additionally, we looked for orthologs of known susceptibility (*S*) genes targeted by *Xoc* or *Xoo* TALEs (e.g., SWEETs, OsSULTR3;6) and queried whether they were among the top predictions for *Xol* TALEs. No known *Xoc* or *Xoo* target homolog was found. It is, however, possible, that while not targeting the direct orthologs of these *S* genes, *Xol* TALEs may be inducing similar functions, since the predicted targets contain genes annotated with similar functions to known *S* genes including sulfate transporters, nodulins and various families of transcription factors (Supplementary Table S3). Expression data and further experiments are necessary to effectively identify the biological mechanisms of these TALEs.

## Discussion

*Xanthomonas oryzae* pv. *leersiae*, which has been isolated from the pervasive weed species *L. hexandra* surrounding rice paddies (Fang et al., 1957; Wonni et al., 2014), was historically grouped as a distinct species and pathovar (Vauterin et al., 1995; Triplett et al., 2011; Wonni et al., 2014). We compared pathogenicity of multiple strains on diverse cereal hosts and complete genomes of five *Xol* strains from Burkina Faso, China, Mali, and Uganda. Similar to *Xoc*, *Xol* strains caused water soaked lesions on rice and *L. hexandra*, but were not virulent to wheat and barley. ANI is a widely accepted baseline beyond DNA–DNA hybridization for taxonomic placement of prokaryotes into a species, not a pathovar, at a threshold of >95–96% (Konstantinidis and Tiedje, 2005; Goris et al., 2007; Richter and Rossello-Mora, 2009; Kim et al., 2014; Bull and Koike, 2015). In phylogenetic analyses, these five *Xol* strains, representing geographic and temporal diversity, grouped more closely with *Xo* pathovars than other members of this genus, and were above a species delineation threshold in ANI analyses. Therefore, we propose re-naming these strains from *Xcl* to *Xol* (Fang et al., 1957) comb. nov.

*Xanthomonas oryzae* pv. *leersiae* colonize and cause water-soaking on rice leaves, but the southern cutgrass isolates are not as aggressive as *Xo* isolated from rice. The lesions caused by *Xol* were phenotypically similar to rice BLS caused by *Xoc* and these strains did not cause disease when introduced into rice or southern cutgrass by leaf-clipping. Thus, we suggest that *Xol* are not systemic pathogens, and are more like *Xoc* than the systemic relative *Xoo*.

T3Es, as important contributors to bacterial pathogenicity, may define host range, and can also inform lineages and evolutionary relationships among different populations of related bacteria (Arlat et al., 1991; Hajri et al., 2012; Wonni et al., 2014; Schwartz et al., 2015). Studies in *X. oryzae* have shown that different lineages are shaped by T3E repertoires and reflect phenotypic adaptation to their agroecosystems (Hajri et al., 2012; Quibod et al., 2016). Likewise, this study sought to uncover similarities between *Xo* and *Xol* effector repertoires that could further inform their evolutionary lineage. Out of a set of previously defined core effectors for *X. oryzae* [*avrBs2*, *avrBs3 (TALEs)*, *xopL*, *xopN*, *xopP*, *xopQ*, *xopV*, *xopW*, *xopY*, *xopAA*, *xopAB*, *xopAE*, *and xopF1*) (Hajri et al., 2012), *Xol* strains contain all except *xopW*, which is absent in three of the five strains. Although present in strains BAI23 and NJ611, *xopW* contains a large IS. This IS most likely prevented its amplification in previous studies (Wonni et al., 2014). Interestingly, this same IS was identified in other African *Xoc* strains, consistent with a shared evolutionary origin with *Xol* (Hajri et al., 2012). On the other hand, *xopD* is present in *Xol* but absent in other *X. oryzae.* XopD is a SUMO protease mimic that suppresses host defense responses during *Xanthomonas euvesicatoria* infection (Kim et al., 2008, 2013). In addition to *Xol*, *xopD* is predicted to be present in *X. campestris* pv. *campestris*, *X. euvesicatoria*, and *Acidovorax citrulli*^4^. Absence in other *Xo* suggests an independent acquisition by *Xol.* Conversely, loss of this effector at some time by *Xoo* or *Xoc* could have occurred, but further validation of these hypotheses is necessary. Since it is not known what level of virulence, and/or host specificity any T3E conveys for *Xol*, future work should include functional validations.

We also assessed TALE diversity in *Xol* strains, using genomic assemblies based on long reads generated using SMRT sequencing. While according to phenotyping, phylogenomics, and non-TALE Type III effector repertoires, *Xol* most closely resembles *Xoc*, the *Xol* TALE sequences more closely resemble African *Xoo. Xol* and African *Xoo* also possess, on average, smaller TALomes (TALEs per genome) than *Xoc* or Asian *Xoo.* Furthermore, their TALEs also have less diverse repeat arrangements as evidenced by shorter genetic distances in pairwise TALE repeat alignments. A possible explanation for this feature is that TALEs from *Xol* and African X*oo* more closely resemble the ancestral TALE repertoire for the group and have not undergone the extensive diversification found in *Xoc* and Asian *Xoo.* A hypovirulent strain of *Xoc* was isolated from rice in the Yunnan province of China that also contains nine TALEs. This strain was used in heterologous expression assays to determine targets and function of Tal7 (Cai et al., 2017). Unfortunately, the TALome of this strain is unavailable at this time and it is unclear if this strain is related to the *Xol* strains characterized in this study.

The expansion of *Xoc* and *Xoo* TALomes is curious since many of the TALEs they carry seem not to be required for virulence and some may even have redundant functions (Pérez-Quintero et al., 2013; Cernadas et al., 2014). Given their repetitive nature and a general tendency toward homogenization of repeats, TALEs have been proposed as being selected for evolvability, that is, selected for their ability to quickly recombine (Schandry et al., 2016). Having an expanded TALome would further allow frequent recombination and generation of new TALE variants, as reflected in bigger evolutionary distances between repeat arrangements. This selection of a bigger TALome has been proposed to be driven by extensive breeding for resistance in the host plant (Schandry et al., 2018). When exposed to a resistant host population, a pathogen population can benefit from carrying a heterogeneous and redundant set of effectors, since preexisting isolates harboring a set advantageous for the new conditions would then be selected, in what has been proposed as a type of evolutionary “bet-hedging” strategy (Win et al., 2012). It is then possible that *Xoc* and Asian *Xoo* have historically encountered more resistance, possibly related to domestication of its primary host, than *Xol* and African *Xoo*, leading to a selection for expanded TALomes.

At least two resistance genes (*Xa1* and *Xo1*) in *O. sativa* specifically recognize TALEs (Ji et al., 2016; Triplett et al., 2016), and *Xoc* and *Xoo* seem to have benefited from their expanded TALomes by selecting TALE variants (iTALEs or truncTALEs) that can specifically suppress resistance mediated by these genes (Ji et al., 2016; Read et al., 2016). Meanwhile *Xol* infecting *L. hexandra*, as African *Xoo* originally infecting *Oryza glaberrima* (Gonzalez et al., 2007), may have never encountered similar resistance in its host, and thus lacks these TALE variants and cannot cause disease on varieties carrying *Xo1* (Figure 1).

TALome expansion may have been accompanied by, or be a consequence of, higher genome plasticity in the *X. oryzae* clade as evidenced by a high amount of genome and gene duplication and a high frequency of IS elements, these measures being generally lower in *Xol*. Within the clade, differences may be once more related to resistance in the host, with the more plastic genomes (Asian *Xoo*) matching more variable host populations. The requirement of plastic genomes in the clade as a whole has been hypothesized to be a consequence of rice cultivation through millennia (Salzberg et al., 2008; Bogdanove and Voytas, 2011), and suggest the ancestor of the clade faced an already variable population. Which then raises the question of where *Xol* falls within this scenario of adaptation to a cultivated crop?

Rice and southern cutgrass are closely related members of the *Poaceae. Leersia* species are often used as an outgroup in phylogenetic and, most recently, genomic investigations (Copetti et al., 2015). Evidence of genome duplication events and defense response genes shared by rice and *Leersia* species has been reported (Jacquemin et al., 2009; Xiao et al., 2009). It is plausible that based on their genetic and evolutionary relatedness that respective pathogens of *Oryza* and *Leersia* evolved independently. The US strains of *Xo*, which do not contain intact TALEs, could have been progenitors of other *Xo* pathovars, having acquired TALEs over time to enhance virulence on rice or *Leersia* spp. (Triplett et al., 2011). Significant trade between the United States, Africa, and Asia could allow for movement of strains across continents. However, it is not clear if *Xol* is currently present in the United States, despite early reports of *L. hexandra* being an alternate host for *Xoo* (Gonzalez et al., 1991). Alternatively, this system may represent a sympatric scenario where *Xoo*, *Xoc*, *Xo*, and *Xol* all lived in the same habitat, providing the opportunity to exchange genetic material and to adapt to their respective host while maintaining basic homology (Jacques et al., 2016). In this scenario, given its similar infection biology and overall similarities to *Xoc, Xol* may be a specialized subgroup originating from a *Xoc*-like population able to colonize southern cutgrass, or vice versa.

How specifically adapted *Xol* strains are to either *L. hexandra* or rice remains a fascinating question, since *Xol* could potentially represent an emerging pathogen for rice. Here we have shown that *Xol* can, to some extent, infect rice, and two of the isolates sequenced in this work were originally recovered from symptomatic rice leaves in a field (BB 151-3 and BB 156-2). To get insights into host adaptation, we attempted to predict targets for *Xol* TALEs in *L. hexandra* using *L. perrieri*, the only available genome from the genus, as proxy and *O. sativa*. Overall, the predictions indicate that *Xol* TALEs induce different sets of genes than other *X. oryzae*, and that different genes are induced in rice and *Leersia* sp. given that relatively few orthologues are predicted as targets in both genomes. Of particular interest, given the phenotypic similarities with *Xoc*, is the predicted targeting of genes annotated as sulfate transporters. Four genes in the *L. perrieri* genome corresponding to orthologs of sulfate transporters from rice (LOC_Os03g09940, LOC_Os03g09970, LOC_Os03g09980, and LOC_Os09g06499) were predicted to be targeted by TALEs from at least one *Xol* strain with high prediction scores (Supplementary Table S3). The primary virulence target of Tal2g from *Xoc* is *OsSULTR3;6* which is a member of the sulfate transporter family 3 (Takahashi et al., 2011; Cernadas et al., 2014). It is feasible that the TALEs targeting sulfate transporters in *Leersia* mirror the virulence function of Tal2g. However, transcriptomic data and further biological validation are required since it is still possible that the few-shared genes in the predictions are true targets and similar functions are required for virulence in both hosts.

## Conclusion

In summary, we propose *Xol*, which was isolated from rice and southern cutgrass (*L. hexandra*), a weedy grass closely related to rice, as a new member of the *X. oryzae* species. Genomic analysis and disease phenotyping on various hosts demonstrated the close relationship of *Xol* to the rice pathogens *Xoo* and *Xoc.* T3E and TALE content of the *Xol* indicated that this group of organisms uses similar virulence mechanisms to the rice pathogens. While weeds such as southern cutgrass are not agronomic crops, they are competitors for resources and potential reservoirs of pathogen inoculum, they are important in management considerations for rice growers. Interfering in any agroecosystem requires comprehensive consideration. Certain *Leersia* sp. are used as banker plants for the critically important rice brown plant hopper (Zheng et al., 2017), therefore integrated management of weeds surrounding rice paddies will require prospecting and balance of all possible pests. The fact that they harbor a pathogen group that can also impact rice emphasizes that, in general, more attention should be focused to the surrounding ecosystem in rice production and more broadly in any crop rotation as a general management strategy. Research contributing toward understanding the *Xol/*rice/southern cutgrass pathosystem will be significant for all rice-producing countries.

## Author Contributions

JML, AP-Q, RK, BS, VV, and JEL conceived and designed experiments. JML, AP-Q, RK, ED, and JJ performed the experiments. SS, HD, IK, RO, OK, and VV collected and provided new *Xol* strains. JML, AP-Q, RK, and JZ analyzed the data. RK, RO, OK, BS, VV, and JEL provided resources and supervision. JML, AP-Q, BS, VV, and JEL developed the manuscript.

## Conflict of Interest Statement

The authors declare that the research was conducted in the absence of any commercial or financial relationships that could be construed as a potential conflict of interest.

## Footnotes

1 www.isppweb.org/about_tppb_naming.asp

2 https://github.com/thackl/minidot

3 https://github.com/alperezq/experimenTAL

4 www.xanthomonas.org
