# Supplementary figures and images for: A Pathovar of Xanthomonas oryzae Infecting Wild Grasses Provides Insight Into the Evolution of Pathogenicity in Rice Agroecosystems
Source: Front Plant Sci. 2019 Apr 30;10:507. doi: 10.3389/fpls.2019.00507 (PMC6503118; doi:10.3389/fpls.2019.00507)

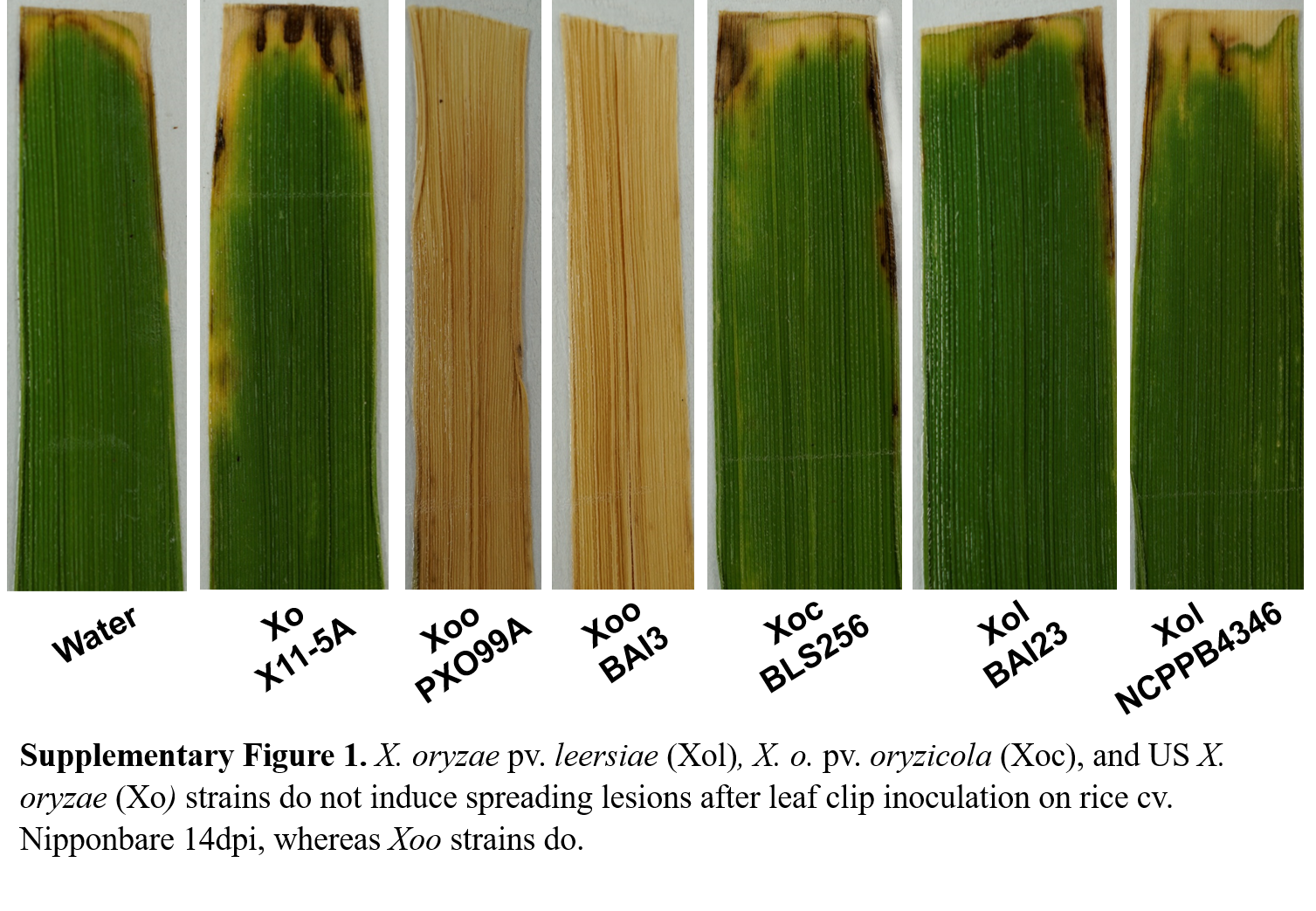

Supplement: Supplementary file 1 [file Image_1.TIF]

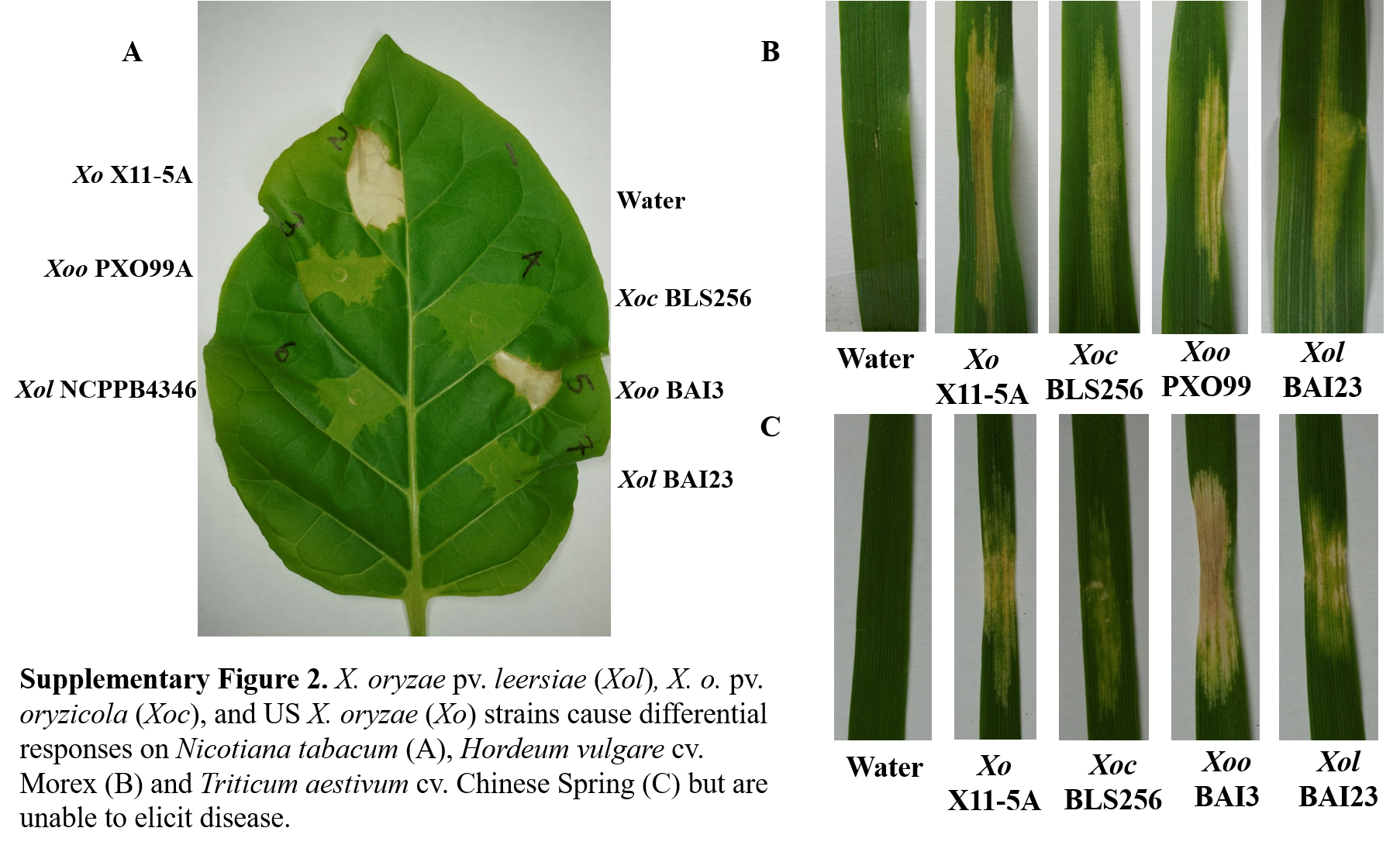

Supplement: Supplementary file 2 [file Image_2.tif]

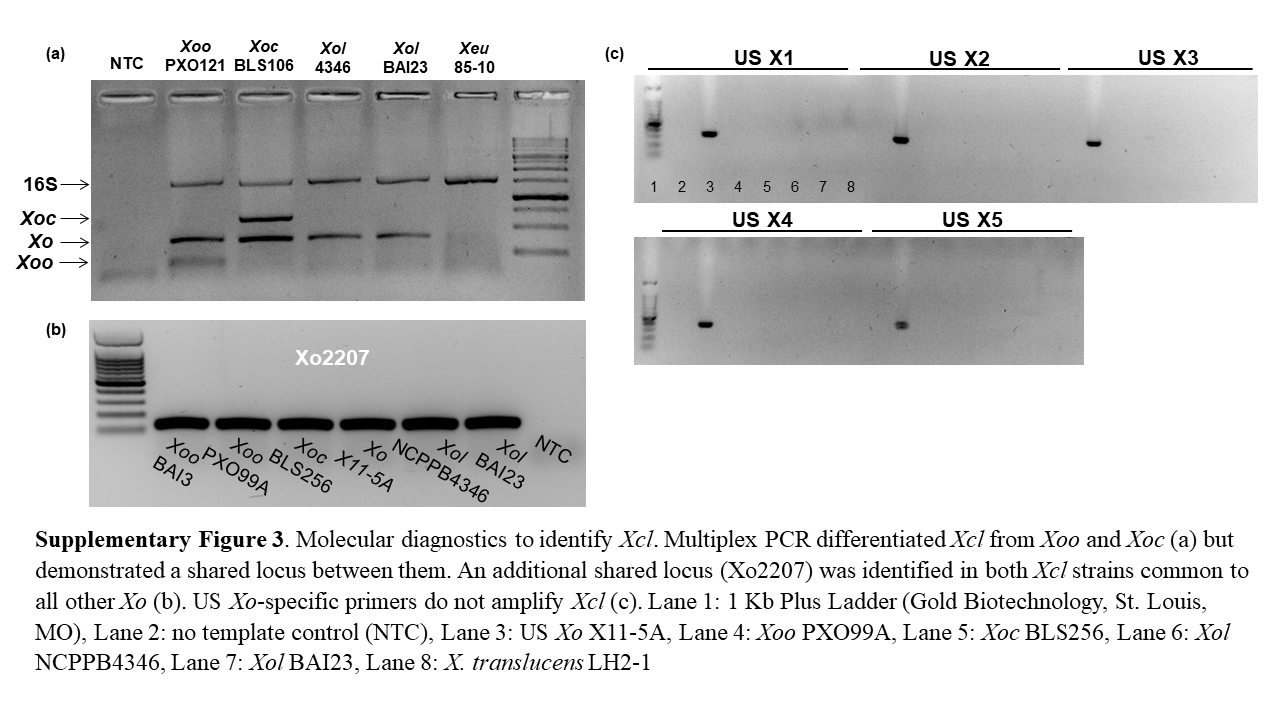

Supplement: Supplementary file 3 [file Image_3.tif]

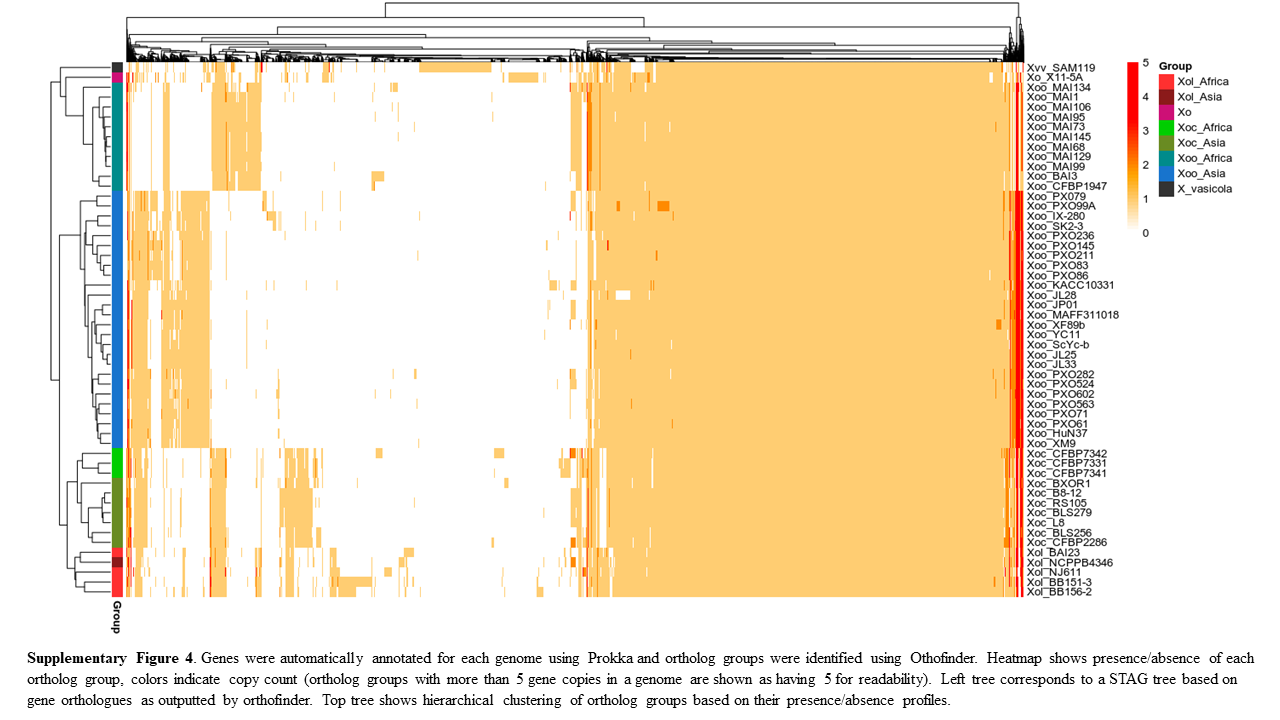

Supplement: Supplementary file 4 [file Image_4.tif]

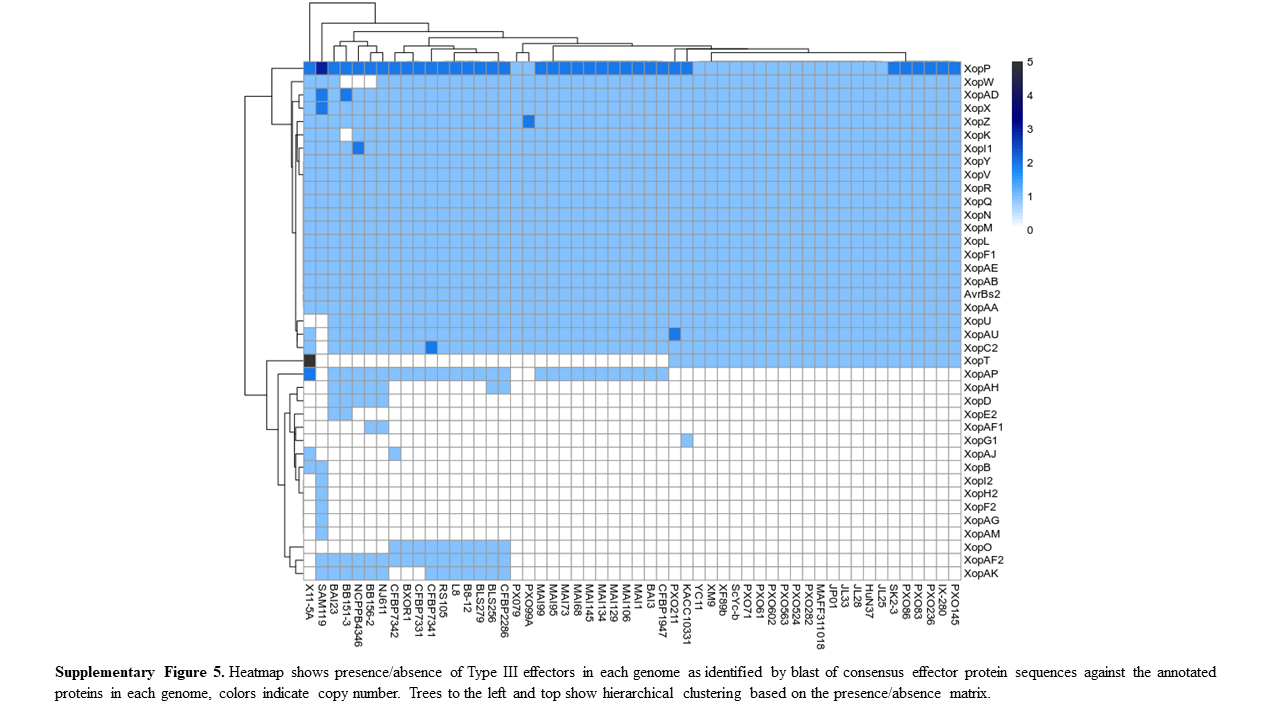

Supplement: Supplementary file 5 [file Image_5.tif]

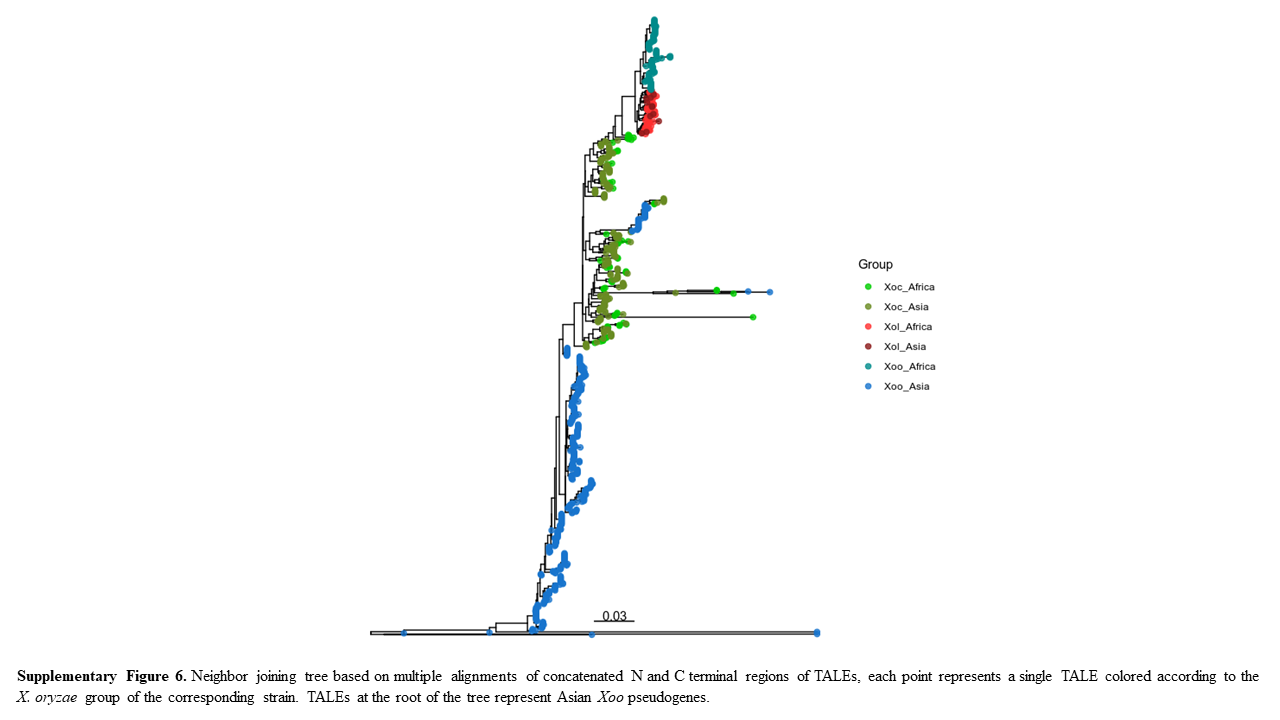

Supplement: Supplementary file 6 [file Image_6.tif]

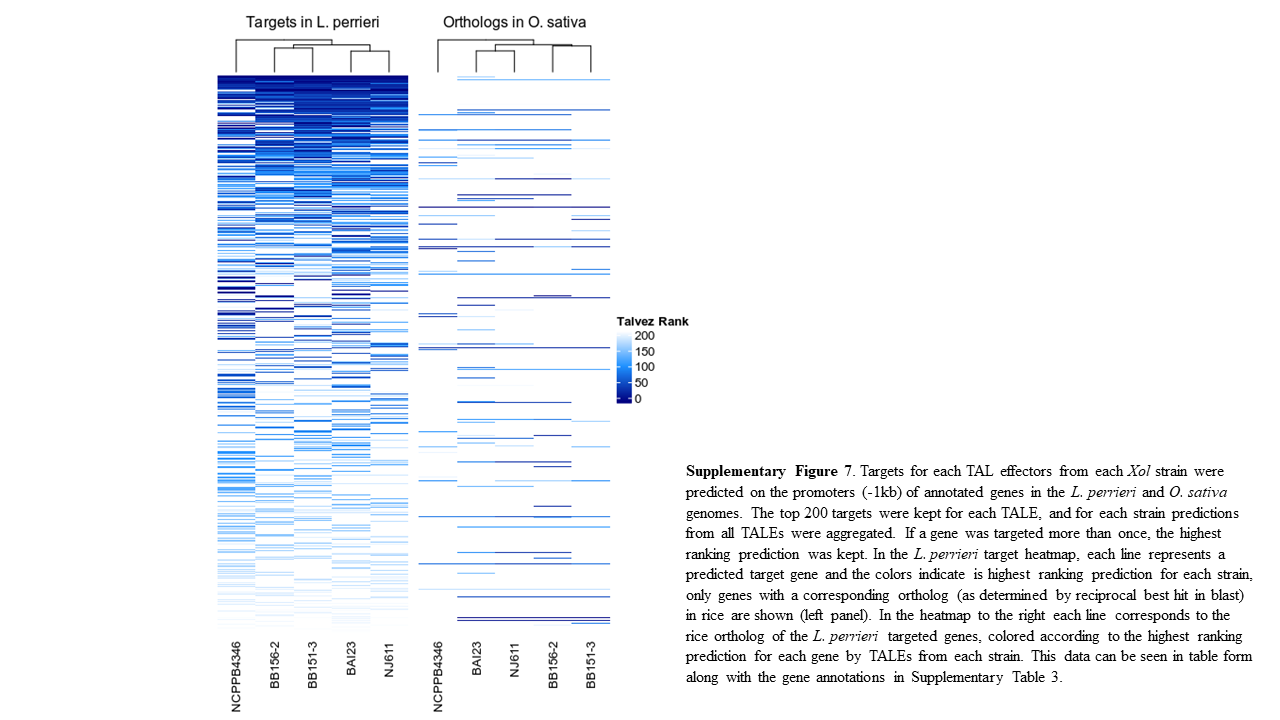

Supplement: Supplementary file 7 [file Image_7.tif]
