# Supplementary material for: A Pathovar of Xanthomonas oryzae Infecting Wild Grasses Provides Insight Into the Evolution of Pathogenicity in Rice Agroecosystems
Source: Front Plant Sci. 2019 Apr 30;10:507. doi: 10.3389/fpls.2019.00507 (PMC6503118; doi:10.3389/fpls.2019.00507)
Supplement: Supplementary file 8 [file Table_1.DOCX]

**Supplementary Table 1.** Primers developed with specificity to *X. o.* pv. *leersiae*.

| **Name** | **Sequence (5’-3’)** | **Product size (bp)** | **Annealing Temp.** |
| --- | --- | --- | --- |
| Xol5_F | TGGTTGTCTGTTTTGGTTCG | 206 | 52 ºC |
| Xol5_R | TGCAAACGGATGTGCATAGT |  |  |
| Xol7_F | TTGCTACGGTATCCGAAAGG | 200 | 52 ºC |
| Xol7_R | CGAAGTATGCAACGGCCTAT |  |  |
